# Properties of an Antimicrobial Molecule Produced by an *Escherichia coli* Champion

**DOI:** 10.3390/antibiotics9010006

**Published:** 2019-12-21

**Authors:** Sarah-Jo Paquette, Tim Reuter

**Affiliations:** 1 Alberta Agriculture and Forestry, #100-5401 1st Ave. South, Lethbridge, AB T1J 4V6, Canada; sarahjo.paquette@uleth.ca; 2 Department of Biological Sciences, University of Lethbridge, 4401 University Drive West, Lethbridge, AB T1J 4V6, Canada

**Keywords:** *E. coli*, Shiga toxin, inhibition, heat resistance, pH resistance, enzyme resistance, microcins, antimicrobial molecule

## Abstract

Over recent decades, the number and frequency of severe pathogen infections have been increasing. Pathogen mitigation strategies in human medicine or in livestock operations are vital to combat emerging arsenals of bacterial virulence and defense mechanisms. Since the emergence of antimicrobial resistance, the competitive nature of bacteria has been considered for the potential treatment or mitigation of pathogens. Previously, we identified a strong *E. coli* competitor with probiotic properties producing a diffusible antimicrobial molecule(s) that inhibited the growth of Shiga toxin-producing *E. coli* (STEC). Our current objective was to isolate and examine the properties of this antimicrobial molecule(s). Molecules were isolated by filter sterilization after 12 h incubation, and bacterial inhibition was compared to relevant controls. Isolated antimicrobial molecule(s) and controls were subjected to temperature, pH, or protease digestion treatments. Changes in inhibition properties were evaluated by comparing the incremental cell growth in the presence of treated and untreated antimicrobial molecule(s). No treatment affected the antimicrobial molecule(s) properties of STEC inhibition, suggesting that at least one molecule produced is an efficacious microcin. The molecule persistence to physiochemical and enzymatic treatments could open a wide window to technical industry-scale applications.

## 1. Introduction

At a certain point in time, humans triggered an evolutionary Big Bang by shaping a new microbial multiverse. Ancestral humans were “hunter-gatherers”, living in small nomadic communities foraging for food. Later those communities transitioned to settle permanently in one place, cultivating food and resources. This was the origin of forming areas with dense populations of humans and livestock [1,2]. Simultaneously, microbial communities adapted, competed, evolved, and proliferated within those close interactions across agriculture and humans alike. Commensal or virulent *Escherichia coli* were some of the species among them. The emergence of lethal diseases (e.g., as early as pandemic reports of the Justinian Plague or Black Death) carried by host-adapted pathogens could only be sustained in areas of dense human populace [2]. These environments are also prime spaces to foster bacterial competition for the existence, often coined as the “survival of the fittest” [3,4,5,6].

Competition can be categorized as exploitative or interference-based [7]. Exploitative interactions are those where one bacterium is using nutrients more efficiently, effectively starving a competitor. However, exploiter champions can be out-competed by a weaker exploiter using interference competition. Interference competition is based on the production of antagonistic factors that eliminate competitors using contact-dependent (growth inhibition and Type VI secretion systems), or contact independent (bacteriocins and other diffusibles, such as antibiotics) means [3,8].

Bacteriocins are molecules (proteins and peptides) produced by both Gram-positive and Gram-negative bacteria [9] and are most often inhibitory to close relatives only [10,11]. However, some Gram-positive bacteriocins have been shown to combat Gram-negative bacteria [12]. Gram-negative bacteriocins have been studied and identified in *E. coli* and are coined colicins and microcins [13]. Colicins are high-molecular-mass (30–80 kDa) proteins [12] and tightly controlled by the bacterial SOS system [4,14]. In contrast, microcins, peptides with molecular masses below 10 kDa, are protease and temperature resistant and are reported to resist pH extremes [12,15]. These characteristics are often associated with bacteriocins of lactic acid bacteria [12].

Bacteriocins are being considered as potential agents to replace antibiotics and have been suggested for use in humans and in livestock as a method to mitigate pathogens [16]. Bacteriocins are considered “agents of competition” [10] and represent a microbial strategy to out-compete their rivals [4,12]. Bacteriocins are unique compared to traditional antibiotics, as they harbor a restricted killing spectrum, targeting specific bacteria or species [10,16]. The targeted killing of specific bacteria makes producers of these bacteriocins an ideal probiotic. In fact, discovered 100 years ago, Mutaflor^®^ is a commercially available probiotic that contains the strain *E. coli* Nissle which produces two microcins, which are thought to be crucial in the ability of this strain to antagonize *E. coli* and *Salmonella* pathogens [17,18,19].

Among bacterial pathogens, Shiga toxin-producing *E. coli* (STEC) produces a potent toxin (Shiga toxin), which, in conjunction with other factors, causes severe, often foodborne infections in humans [20,21]. Various mitigation strategies for this pathogen have been considered, including vaccines [22], direct-fed microbials [23], and tannins [24], but none have consistent efficacy. Another mitigation strategy being considered to control STEC is using probiotic bacteria to competitively eliminate the pathogens [25], as demonstrated by the effective use of *E. coli* Nissle to alleviate intestinal infections in humans [17]. Likewise, a colicin-producing *E. coli* isolated from sheep fecal samples was shown to inhibit the STEC O157:H7 [25].

In a previous study in our laboratory, we demonstrated that various *E. coli* strains isolated from beef cattle feces produce diffusible molecules capable of affecting competitor growth when separated by a 4 to 10 mm barrier of agarose [26]. However, the strength of these molecules varied among strains, which led to the identification of a strong competitive non-pathogenic *E. coli* champion that produces a diffusible molecule(s) capable of out-competing 31 different *E. coli* strains including STEC O26, O111 and O157 [26].

The objective of this project is to characterize further the diffusible molecule(s) produced by this *E. coli* champion, while investigating the physicochemical and biological properties of the molecule as well as its antimicrobial potential.

## 2. Materials and Methods

### 2.1. Bacterial Strains: Cultures, Media and Culture Conditions

Both *E. coli* strains used in this study, O157A and O103F, were described previously [26]. Briefly, *E. coli* strains were streaked from glycerol stocks onto MacConkey Agar (MAC, BD, Sparks, NV, USA). Plates were incubated overnight (16–18 h) at 37 °C. A single colony was selected from each plate and inoculated into *E. coli* broth (EC, EMD Millipore, Etobicoke, ON, Canada) and incubated overnight at 37 °C with shaking at 150 rpm. Overnight cultures of O157A (STEC) and O103F (no detected virulence genes) were diluted to an optical density (OD) of 0.1 measured at a wavelength of 600 nm in fresh EC and grown for 3 h. The 3 h culture was then used as inoculation for the treatments.

### 2.2. Molecule Isolation Assay

Molecule isolation assay was adapted from Kulp and Kuehn, 2010 [27] by following the isolation protocol for natural outer membrane vesicles up to and including step 2. O103F molecule is the cell-free supernatant collected after 12 h of growth of O103F in EC, containing AntiMicrobial Molecule(s) and will subsequently be referred to as AMMO. O157A molecule is the cell-free supernatant collected after 12 h growth of O157A in EC and will be subsequently referred to as SPENT. After isolation, AMMO and SPENT underwent various treatments.

**Figure 1:**
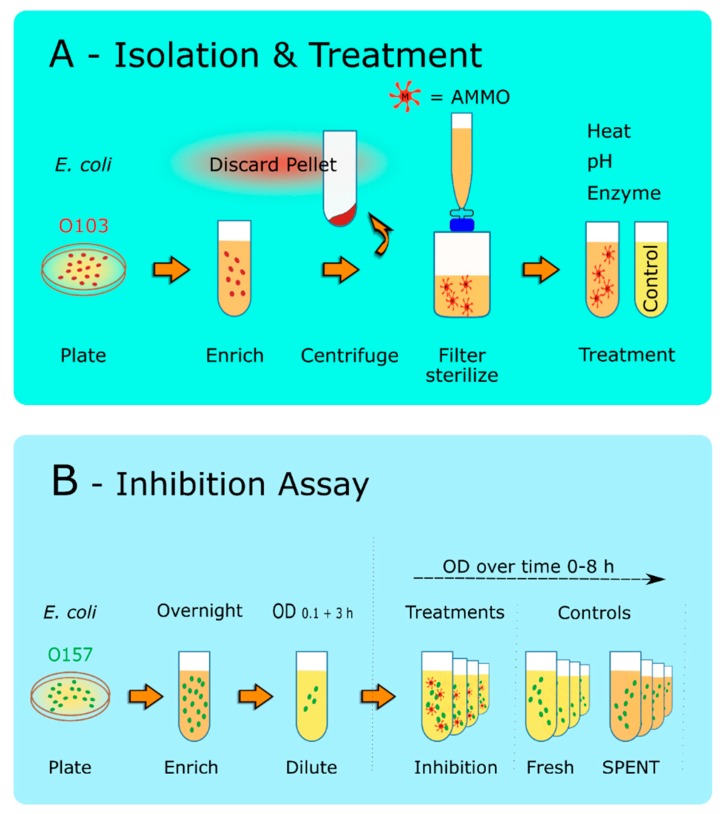
Schematic of the Molecule Isolation Protocol and Inhibition Assay. (**A**)**: Isolation** and **Treatment**. *E. coli* O103F was grown overnight on a MacConkey Agar plate, and a single colony was grown in EC for 12 h. Cells were pelleted at 10,000× *g* for 10 min. The cell pellet was discarded, and the supernatant was filter-sterilized using a 0.22 µm filter. The sterilized supernatant was then treated to examine, heat, pH, or protease digestion. Note: SPENT was also prepared using the same protocol. (**B**)**:** **Inhibition Assay**. O157A was grown overnight for 12 h in EC. The cells were then diluted to an OD_600nm_ of 0.1 and grown for 3 h. This culture was then used to inoculate the AMMO and the controls. OD_600nm_ measurements were taken at 0, 2, 4, 6, and 8 h. **Note:** (**1**) O103F was also prepared using the same protocol when utilized in the experiment. (**2**) AMMO is the cell-free supernatant collected after 12 h *E. coli* O103F growth. SPENT is the cell-free supernatant collected after 12 h *E. coli* O157A growth.

Both *E. coli* (O103F and O157A) cultures were grown individually for 12 h and centrifuged (10,000× *g*) for 10 min to prepare the supernatant (Figure 1). The supernatant was then filter-sterilized using a 0.22 µm filter (Pall Life Sciences, Ann Arbor, MI, USA) to remove all bacterial cells. The O103F supernatant (AMMO) was added to fresh EC to test the growth of O157A cells during the inhibition assay. Each experiment had a complete set of four controls to measure the effect of AMMO. The first control, O157A, was diluted to a starting OD of 0.1 in a final volume of 5 mL of fresh EC to demonstrate regular/healthy growth of *E. coli* O157A in fresh media. The second control, O157A, was diluted to a starting of OD 0.1 in a total of 5 mL (3.75 mL SPENT and 1.25 mL culture and fresh EC) to take into account the effect of depletion of nutrients and metabolic end products in the isolated supernatants on O157A growth. The third and fourth controls, 3.75 mL of the supernatants (AMMO or SPENT), were each individually added to 1.25 mL fresh EC to ensure that filter-sterilization was successful, and all live cells were removed from the supernatants (AMMO and SPENT).

#### 2.2.1. Isolation Confirmation

A culture of either O157A or O103F was diluted to a starting OD of 0.1 in a final volume of 5 mL (3.75 mL of AMMO or SPENT and 1.25 mL culture and fresh EC). A second control was prepared with O157A or O103F diluted into fresh EC. Subsequently, all cell preparations were incubated at 37 °C at 150 rpm for 24 h. OD measurements were taken at 0, 2, 4, 6, 8, and 24 h to extrapolate cell densities using an initially determined strain-specific growth curve data (slope equation: O157A → y = −6 × 10^6^ × 2 + 4 × 10^7^ × −2 × 10^6^ and O103F → y = −2 × 10^7^ × 2 + 7 × 10^7^ × −3 × 10^6^).

The growth curve data were analyzed by comparing strain-specific OD values versus CFU plate counts in parallel at the time point 0, 2, 4, 6, 8, and 24 h (data not shown). All experiments with AMMO and SPENT supernatants were conducted likewise, following the treatments as described below. All experiments were replicated on alternate days with fresh cultures, AMMO, and SPENT.

#### 2.2.2. pH Treatment

To examine the effect of pH on AMMO, hydrochloric acid (HCl) or sodium hydroxide (NaOH) was added to the supernatants containing the isolated AMMO or SPENT to lower or increase the pH of the solutions to 3 or 11, respectively. The supernatants were then incubated at either pH for 3 h. After the incubation, the supernatants (AMMO and SPENT) pH were neutralized to the pH pre-treatment by titrating either HCl or NaOH solutions and subsequently used for the inhibition assay (Figure 1B).

#### 2.2.3. Autoclave Treatment

To examine the effect of heat and pressure treatment, the prepared AMMO and SPENT supernatants were autoclaved for 20 min at 121 °C and 18 psi. After cooling to room temperature, the supernatants were subsequently used for the inhibition assay (Figure 1B).

#### 2.2.4. Trypsin Treatment

To examine the effect of trypsin (Calbiochem, La Jolla, CA, USA) digestion, 5 µL of a prepared trypsin solution (7.5 units/µL) was added to 20 mL of the prepared AMMO and SPENT supernatants and incubated for 3 h at 37 °C. After the incubation, trypsin digestion was stopped by heating the mixture for 10 min at 95 °C. After cooling to room temperature, AMMO, and SPENT supernatants were used for the inhibition assay (Figure 1B).

#### 2.2.5. Chymotrypsin Treatment

To examine the effect of chymotrypsin (Sigma-Aldrich, Oakville, ON, Canada) digestion, 4 µL, n or 40 µL (1 unit/µL) of prepared chymotrypsin solution was added to 20 mL of the prepared AMMO and SPENT supernatants. The supernatants were then incubated for 3 h at room temperature. After the incubation, chymotrypsin digestion was stopped by heating the mixture for 10 min at 80 °C. After cooling to room temperature, AMMO, and SPENT supernatants were used in the inhibition assay (Figure 1B).

### 2.3. Analysis of Cell Densities

Extrapolated cell densities of O157A grown in either AMMO or SPENT were examined for the difference in cell numbers between the two supernatants by subtracting the number of cells in the AMMO from the SPENT.

### 2.4. Statistical Analysis

Numerical OD data measured for each experiment were examined for normality and subsequently used for analyses. Time, treatment, control, and interactions were determined for all the experiments using a mixed linear model (Proc Mixed, SAS 9.4, SAS Institute Inc., Cary, NC, USA). *p* values < 0.05 were considered significant. Calculated standard deviations from each experiment are shown as bars within the Figures.

## 3. Results

### 3.1. AMMO Isolation Protocol Confirmation

Preliminary studies for both strains determined that OD measurement corresponded to plate counts in the provided growth curve in Section 2.2.1. OD based measurements were subsequently used to determine cell densities.

**Figure 2:**
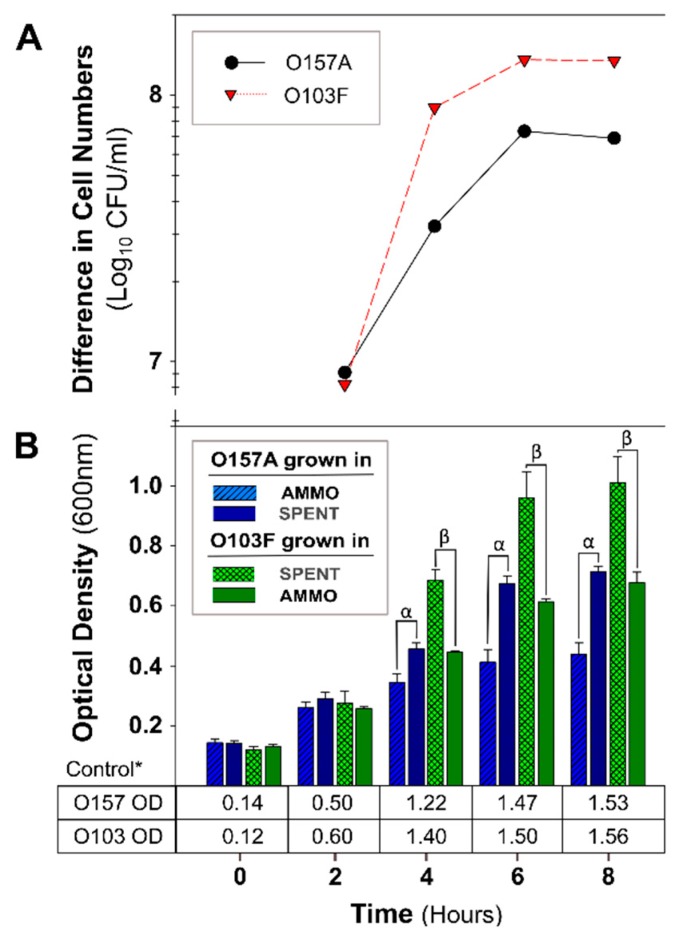
The molecule isolation protocol confirmation results for *E. coli* O157A grown in AMMO and SPENT in comparison to *E. coli* O103F grown in SPENT and AMMO. (**A**): The difference between the cell numbers for either O157A or O103F in the SPENT and in AMMO. (**B**): The OD_600nm_ data for O157A in AMMO, O157A in SPENT, O103F in SPENT, and O103F in AMMO and * control in fresh EC as a numerical value below the bars. Symbols: α and β denote a significant difference between growth in AMMO and SPENT for O157A and O103F, respectively (*p* < 0.05). A comparison of O103F growth in AMMO to O157A growth in SPENT revealed they are not significantly different. **Note:** (**1**) AMMO is the cell-free supernatant collected after 12 h *E. coli* O103F growth. SPENT is the cell-free supernatant collected after 12 h *E. coli* O157A growth. (**2**) Bars are the calculated standard deviation for AMMO and SPENT in each experiment (O157A and O103F) at each time point.

A comparison of OD measurements of O157A grown in a mixture of EC and AMMO to O157A grown in a mixture of EC and SPENT demonstrated that O157A inoculated into AMMO had significantly lower growth at 4, 6, and 8 h (*p* < 0.05) (Figure 2B). Furthermore, the difference in cell density increased over time, and the *E. coli* O157A in SPENT had −6 × 10^7^ CFU/mL more cells than O157A in AMMO at 8 h (Figure 2A). In contrast, the OD of O103F grown in a mixture of EC and SPENT compared to O103F grown in a mixture of EC and AMMO, demonstrated that the O103F cell proliferation was significantly greater (*p* < 0.05) in the presence of SPENT than in AMMO (Figure 2B). Concurrently, the difference in cell density demonstrated that O103F in SPENT had −1.0 × 10^8^ CFU/mL more O103F cells than O103F in AMMO at 8 h (Figure 2A). Furthermore, comparing the growth of O157A in SPENT to O103F in AMMO did not identify a significant difference in growth (*p* > 0.05). Expectedly, all controls of O103F or O157A in fresh EC grew to higher turbidity than O103F and O157A grown in SPENT (Figure 2B).

### 3.2. Investigation of AMMO Properties

Preliminary studies revealed that between 8 and 24 h, AMMO inhibition was diminishing and at 24 h no longer detectable (data not shown). Data are reported for the period up to 8 h.

#### 3.2.1. The Effect of pH, Autoclaving, Trypsin and Chymotrypsin Digestion

**Figure 3:**
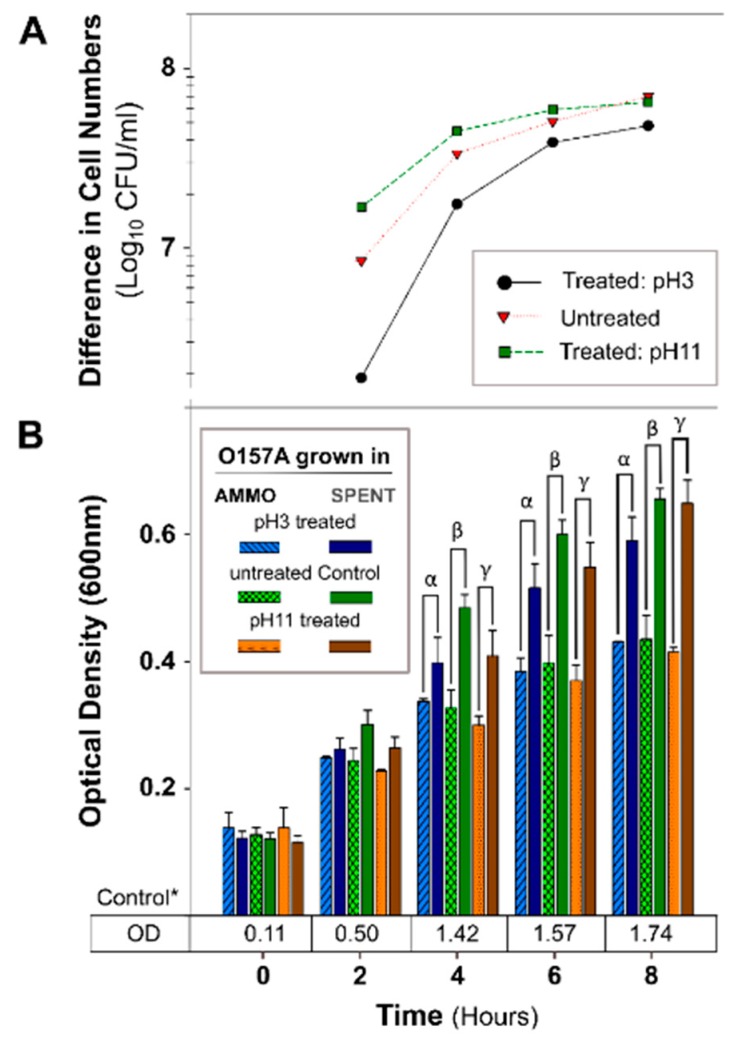
The effect of pH treated AMMO on growth inhibition of *E. coli* O157A at two pH’s 3 and 11. (**A**): The difference between the cell numbers for O157A in SPENT and in AMMO at pH 3, pH 11, and untreated supernatant. (**B**): The OD_600nm_ data for O157A grown in AMMO, in SPENT (pH 3, pH 11, and untreated) and * control in fresh EC as a numerical value below the bars. Symbols: α, β and ɣ denote a significant difference between O157A grown in AMMO and in the SPENT for pH 3, untreated, and pH 11 supernatants, respectively (*p* < 0.05). **Note:** (**1**) AMMO is the cell-free supernatant collected after 12 h *E. coli* O103F growth. SPENT is the cell-free supernatant collected after 12 h *E. coli* O157A growth. (**2**) Bars are the calculated standard deviation for the treated or untreated AMMO and SPENT at each time point.

**Figure 4:**
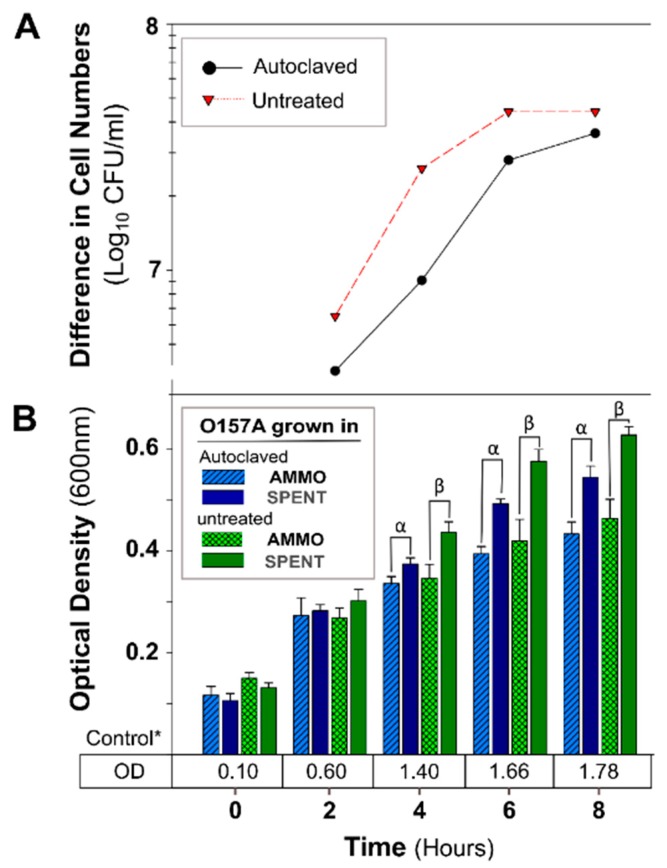
The effect of autoclaving AMMO on growth inhibition of *E. coli* O157A. (**A**): The difference between the cell numbers for O157A in SPENT and in AMMO with treated and untreated supernatant. (**B**): The OD_600nm_ data for O157A grown in AMMO, in SPENT (treated and untreated), and * control in fresh EC as a numerical value below the bars. Symbols: α and β, denote a significant difference between O157A grown in AMMO and in SPENT for treated and untreated supernatants, respectively (*p* < 0.05). **Note:** (**1**) AMMO is the cell-free supernatant collected after 12 h *E. coli* O103F growth. SPENT is the cell-free supernatant collected after 12 h *E. coli* O157A growth. (**2**) Bars are the calculated standard deviation for the treated or untreated AMMO and SPENT at each time point.

**Figure 5:**
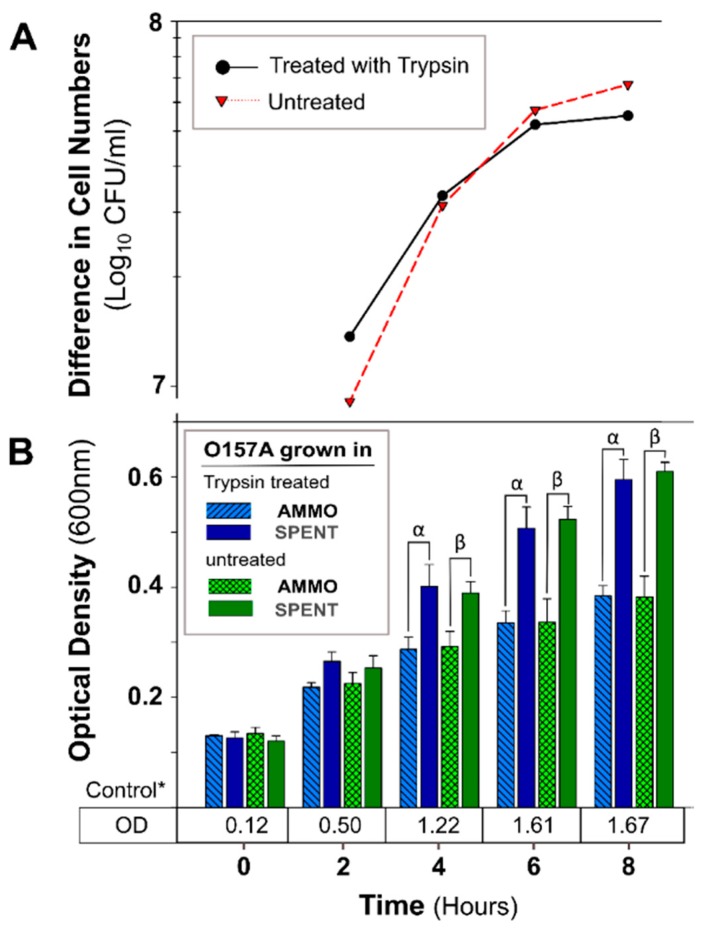
The effect of trypsin protease digestion of AMMO on growth inhibition of *E. coli* O157A over time. (**A**): The difference between the cell numbers for O157A in SPENT and in AMMO treated and untreated supernatant. (**B**): The OD_600nm_ data for O157A grown in AMMO, in SPENT (treated and untreated), and * control in fresh EC as a numerical value below the bars. Symbols: α and β, denote a significant difference between O157A in AMMO and in SPENT for treated and untreated supernatants, respectively (*p* < 0.05). **Note:** (**1**) AMMO is the cell-free supernatant collected after 12 h *E.* *coli* O103F growth. SPENT is the cell-free supernatant collected after 12 h *E. coli* O157A growth. (**2**) Bars are the calculated standard deviation for the treated or untreated AMMO and SPENT at each time point.

**Figure 6:**
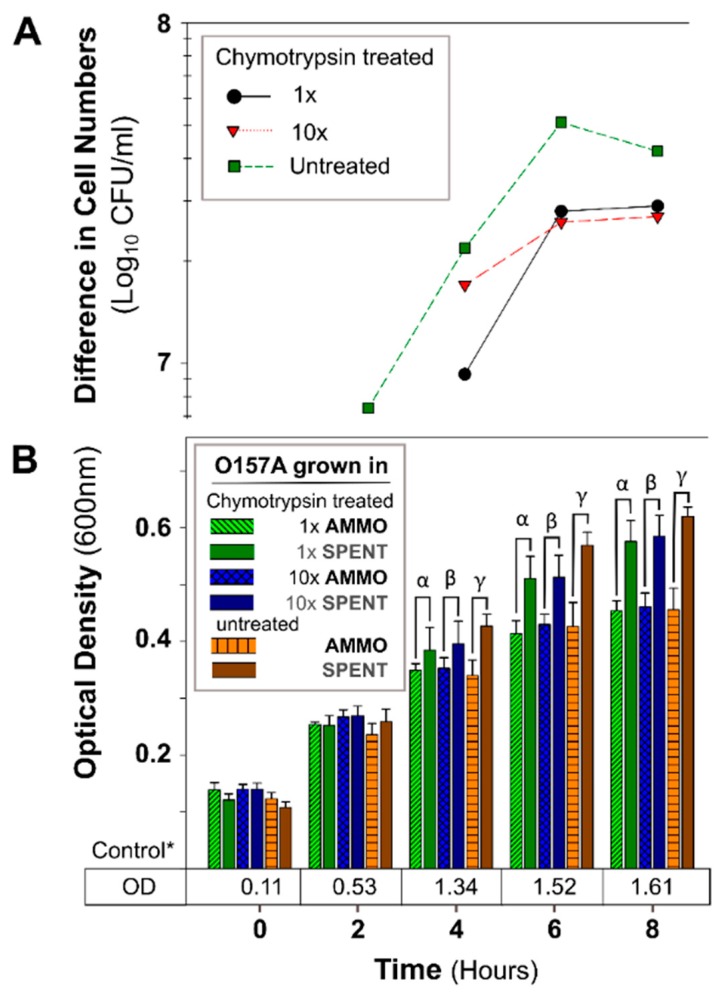
The effect of chymotrypsin protease digestion of AMMO on growth inhibition of *E. coli* O157A over time. (**A**): The difference between the cell numbers for O157A in SPENT and in AMMO with treated and untreated supernatants. (**B**): The OD_600nm_ data for O157A grown in AMMO, in SPENT (treated and untreated), and * control in fresh EC as a numerical value below the bars. Symbols: α, β and ɣ, denote a significant difference between: O157A in AMMO and in SPENT for 1×, 10×, and untreated supernatants, respectively (*p* < 0.05). Time-point = 2 difference in cell number data not shown for chymotrypsin treated AMMO and SPENT (1× and 10×) due to parallel OD_600nm_ data, and the difference is ~zero. **Note:** (**1**) AMMO is the cell-free supernatant collected after 12 h *E. coli* O103F growth. SPENT is the cell-free supernatant collected after 12 h *E. coli* O157A growth. (**2**) Bars are the calculated standard deviation for the treated or untreated AMMO and SPENT at each time point.

The OD of O157A grown in a mixture of EC and AMMO after treatment (pH, Autoclaving, Trypsin, and Chymotrypsin) was not significantly different from O157A grown in EC with untreated AMMO (Figure 3B, Figure 4B, Figure 5B, and Figure 6B). In contrast, a comparison of O157A grown in EC and AMMO (treated or untreated) to the O157A grown in EC and SPENT (treated or untreated) revealed significant inhibition of O157 growth at 4, 6, and 8 h (*p* < 0.05). Additionally, the difference in cell density for treated or untreated AMMO and SPENT increased over time (Figure 3A, Figure 4A, Figure 5A and Figure 6A) with the largest difference in cell density at 8 h (Table 1). The pure culture control of O157A in fresh EC grew to higher turbidity than O157A grown in SPENT in each experiment (Figure 3B, Figure 4B, Figure 5B, and Figure 6B).

#### 3.2.2. Comparison of Inhibition Activity

**Figure 7:**
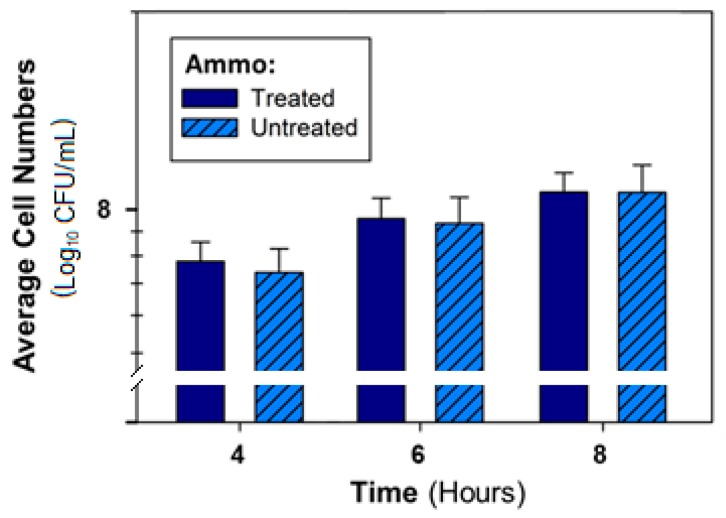
Comparison of average inhibition activity against *E. coli* O157 of treated AMMO across all treatments versus untreated AMMO (control) at time point 4, 6, and 8 h. **Note:** (**1**) Bars are the calculated standard deviation for the treated or untreated AMMO, respectively, at each time point. (**2**) AMMO is the cell-free supernatant collected after 12 h *E. coli* O103F growth.

The comparison of the average inhibition activity of AMMO across the different treatments at 4, 6, and 8 h to untreated AMMO further revealed no effect of treatment on inhibition (Figure 7). Variability between the treated and untreated AMMO is minor, as demonstrated by the standard deviation, shown as bars on the graph.

## 4. Discussion

Interference competition is either contact-dependent or contact independent. Due to the removal of viable bacterial cells in our study, the inhibition of the competitor growth was solely based on contact-independent mechanisms. Contact independent inhibition occurs by one of the following known mechanisms—release of small molecules (less than 10 kDa), proteins (larger than 10 kDa), membrane vesicles, tailocins, and bacteriophages [28]. Across these mechanisms, a striking difference is the sensitivity of the active substances to physiochemical and biological treatments. Except for microcins, none of those known inhibitory agents are reported to persist the combination of extreme heat and pressure (autoclaving). In addition to heat resistance, microcins excreted by Gram-negative bacteria have been reported to be resistant to extreme pH and are resistant to proteolysis [29].

### 4.1. AMMO Isolation Protocol Confirmation

A previous study in our laboratory [26] revealed a competitive O103F champion producing strong diffusible molecule (AMMO), which inhibited the growth of a wide range of STEC isolates, including a STEC runner-up (O157A). Here we isolated and further investigated the AMMO, properties. The trials after isolation confirmed that O157A grown in AMMO was significantly inhibited compared to the O157A grown in SPENT (*p* < 0.05). Additionally, the cell density was greater in SPENT than in AMMO and the difference in cell density between them increased over time. Furthermore, our data are demonstrate that SPENT did not inhibit O103F growth, further confirming our previous results [26], and, in fact, O103F grown in SPENT had a higher cell density than O103F grown in AMMO. Feasibly, O103F has a metabolic advantage and is able to utilize nutrients not used by O157A as the metabolic pathways of different *E. coli* strains have been shown to vary in their ability to utilize different carbon sources [30]. Likely, O103F utilized a remaining nutrient source still present in SPENT. Comparison of O103F grown in AMMO to O157A grown in SPENT did not identify a significant difference in growth in the utilized media, providing evidence that the inhibition effect on O157A growth in AMMO is due to the presence of an antimicrobial compound(s) and not due to a lack of nutrients in the media. In addition, grown in fresh media, O157A and O103F had higher turbidity, demonstrating that inhibition (O157A) or no inhibition (O103F) was due to an external factor (AMMO synthesis) and not due to a strain fitness effect. In confirmation with our previous data, which demonstrated the production of a diffusible antimicrobial through an agarose barrier (no contact between competitors), the isolation of a diffusible and inhibitory AMMO occurred as *E. coli* has been reported to produce colicins and microcins targeting other *E. coli* and close relatives [13,31]. Furthermore, our results revealed that AMMO is effective in the absence of live bacterial cells, which may alleviate regulatory hurdles probiotic bacteria encounter when being considered as a drug to treat human disease [32]. In accordance with previous studies, AMMO inhibited the STEC O157A, and similar effects have been reported for the microcin producing probiotic *E. coli* Nissle, which displaced pathogens from an inflamed gut [19] or reduced the number of STEC O157:H7 and O104:H4 in vitro [33].

Colicin and microcin producers target various systems in their prey and kill them using several mechanisms from forming cell wall channels to the corruption of the intracellular machinery but are themselves resistant to this activity [34,35]. Production of either microcins or colicins is always coupled with resistance genes and antidote synthesis. O157A succumbs to at least one AMMO produced by O103F, suggesting a lack of resistance. In contrast and in accordance with our previous results [26], O103F is not inhibited by any antimicrobial produced by O157A, suggesting O103F is resistant to any antimicrobials produced by O157A. Resistance to colicins and microcins can occur through the production of these antimicrobials since production is always paired with the synthesis of resistance proteins or through mutations in receptors or uptake mechanisms for the bacteriocin [34]. *E. coli* has been shown to produce more than one colicin and microcin [36]. Plasmids encoding bacteriocins are stably maintained in microbial populations, most likely due to the lethal disadvantage of lost resistance [4]. Here, O103F may either have resistance to any O157A antimicrobial because it can produce the antimicrobial or has mutations in the receptors for the O157A antimicrobial as previous research revealed that *E. coli* could acquire resistance to bacteriocins in competition assays [37].

### 4.2. Investigation of AMMO Properties

The results across all trials revealed that AMMO was not affected by any of the treatments. Neither pH, heat (pressure) nor protease digestion affected the inhibitory properties of AMMO. In an applied scenario, the stability of the molecule would offer a wide range of technical treatment options for the implementation of purifications within an industrial-scale setting.

In each trial, there were no significant differences in O157A growth between treated and untreated AMMO, and comparison of the inhibition activity of only the treated AMMO revealed a similar inhibition pattern across all treatments, further demonstrating that treatment had no effect. Additionally, O157A growth was significantly inhibited (*p* < 0.05) when grown in the presence of AMMO compared to SPENT, regardless of treatment, showing treatment did not affect the ability of AMMO to inhibit O157A growth. Concurrently, this effect was further demonstrated when examining cell numbers of O157A, which were more numerous in treated or untreated SPENT compared to treated or untreated AMMO in each experiment. Additionally, pure culture controls of O157A grew to higher cell numbers compared to O157A grown in SPENT, demonstrating that the inhibitory effect was due to the presence of AMMO and not due to any variation of viability. The evidence of physicochemical and enzymatic resistant characteristics of AMMO suggests that at least one microcin is produced by O103F.

Microcins are small antimicrobial peptides and have been mainly discovered in *E. coli* (one in *Klebsiella*) with a molecular weight of less than 10 kDa [38]. Microcins have a narrow killing range, primarily targeting *E. coli* and their close relatives. Remarkably, despite being these “killing machines”, the mode of action of many microcins is unknown, including microcin M, one of the microcins produced by *E. coli* Nissle, a probiotic that has been used for over 100 years to mitigate intestinal pathogens [29]. Microcin properties of extreme pH, protease, and heat resistance [39], are commonly shared with bacteriocins from lactic acid bacteria, [13,38]. Resistance to extreme pH or proteases varies among microcins. Microcin E492 is resistant to low pH [40], while J25 is resistant to both low and high pH extremes [41]. Others are resistant to the protease trypsin but not to chymotrypsin digestion, vice versa or resistant to both [40]. Heat resistance is shared among microcins [40], and microcin J25 has been shown to resist autoclaving (15 min at 121 °C) [41]. The AMMO produced by O103F has analogous characteristics, and plausibly is a molecule of similar design. Furthermore, our data demonstrated that the AMMO effect was no longer detectable after 24 h, an indicator for a “single-use” effect. Microcin C enters the host cells by mimicking nutrient properties and after cell uptake is cleaved by the host intracellular machinery to create the active compound [39]. Plausibly, this cleavage of microcin C is irreversible, and AMMO undergoes a similar transformation.

Aside from those properties discussed above, microcins can be further differentiated from colicins because they are not induced by the bacterial SOS (response to DNA damage) system, and secreted microcins are not lethal to the producing cells [34]. To date, 14 microcins have been identified [12], but only eight have been structurally characterized [29]. Some microcins, such as H47 and I47 or C7 and C51, have similar microcin gene clusters and only differ by 3 or 1 additional genes, respectively [15]. Ultimately, AMMO produced by O103F appears to be a microcin with a change in the microcin gene cluster for an enhanced killing potential. Research comparing the microcin gene cluster of O103F with sequences of known microcins may elucidate such a probability.

The production of microcins by *E. coli* [13] is a tactic used to out-compete their adversaries [4]. Microcins are deemed a potential replacement for antibiotics to mitigate pathogens both in human medicine and in the farm-to-fork continuum [16]. Antibiotic resistance is a global challenge evoked by their overuse in the livestock industry and human medicine, which led to the emergence of resistant pathogens. Contrary to traditional broadband antibiotics, microcins have a narrow, species-specific killing range [10,16]. Since his discovery about 100 years ago in the battlefields of WWI, the *E. coli* Nissle strain has been effectively used to treat human intestinal infections, and the specific Nissle microcins are considered the active antimicrobial substance [17,19].

As a foodborne pathogen, STEC causes severe intestinal infections in humans [20,42], and effective mitigation strategies are lacking [22,23,24]. Use of probiotic bacteria that can out-compete STEC is being considered as an approach to eliminate this pathogen, and colicin producers isolated from sheep fecal samples have been shown to inhibit STEC O157:H7 growth [25].

A previous study in our laboratory showed a diverse STEC growth inhibition by AMMO produced by O103F [26], and the results from this study strongly suggest that O103F produces at least one very effective microcin. Microcins are considered part of the killing repertoire of the probiotic *E. coli* Nissle strain. The type of microcins produced by *E. coli* O103F holds the potential to be used as a STEC mitigation strategy. In logical stepwise approaches, we aim to evaluate this molecule further and gain more knowledge on the antimicrobial properties produced by O103F. Future studies examining the genome and plasmid sequences of O103F for microcins, the biochemical structure of AMMO and physiological properties, including metabolic pathways, are required to elucidate the therapeutic potential of this strain and the antimicrobial it produces.

## 5. Conclusions

Agriculture, as an evolutionary Big Bang, triggered a new microbial multiverse and changed the dynamics of human and microbe (pathogen) interactions. Most recent global transformation, including biological technologies (and the use of antimicrobial substances) have fundamentally altered the way, size, speed, and scope of how we produce and consume food, but in addition, promoted the emergence of virulent microbes with resistance to antibiotics. Microcin molecules are regarded as a potential alternative to antibiotics. Therapeutic microcin properties may warrant the use as a next-generation control strategy in livestock production systems or to mitigate pathogens after human infections. Ultimately, the microcin we tested here inhibited the growth of *E. coli* pathogens after a range of physicochemical and enzymatic inactivation treatments. The antimicrobial properties suggest that this type of isolated molecule could be an antibiotic candidate even in the absence of the viable *E. coli* producer. The resistance to treatments that make the molecule an ideal candidate for industrial-scale isolation and purification technologies.

## Figures and Tables

**Table 1:** Difference in cell density between O157A grown in SPENT versus O157A grown in AMMO at 8 h.

	Supernatant Trials (Cell Numbers in ^x^10^7^)					
Treated	pH 3	pH 11	Autoclaved	Trypsin	C-Trypsin 1x	C-Trypsin 10x
SPENT minus AMMO *	5	6	3	5	3	3
**Untreated Control**	**pH**		**Autoclaved**	**Trypsin**	**Chymotrypsin**	
SPENT minus AMMO *	6		4	6	4	

* Differences in cell density. * Note: In all the tests, the OD_600nm_ of the 12 h culture was measured prior to preparing the supernatant and in each case, both O103F and O157A had similar growth densities at 12 h. Note: (**1**) Difference in cell density is approximate and calculated using previously generated growth curve numerical data for O157A. (**2**) AMMO is the cell-free supernatant collected after 12 h *E. coli* O103F growth. SPENT is the cell-free supernatant collected after 12 h *E. coli* O157A growth. (**3**) C-Trypsin = Chymotrypsin.
